# Research on Grain Food Blockchain Traceability Information Management Model Based on Master-Slave Multichain

**DOI:** 10.1155/2022/7498025

**Published:** 2022-12-27

**Authors:** Yue Li, Xin Zhang, Zhiyao Zhao, Jiping Xu, Zixuan Jiang, Jiabin Yu, Xiaoyu Cui

**Affiliations:** ^1^Beijing Key Laboratory of Big Data Technology for Food Safety, Beijing Technology and Business University, Beijing 100048, China; ^2^Key Laboratory of Internet and Big Data for China Light Industry, Beijing Technology and Business University, Beijing 100048, China; ^3^University of Toronto, St. George Campus, 27 King's College Circle, Toronto M5S 1A1, Ontario, Canada

## Abstract

Aiming at the problems such as slow traceability efficiency, poor sharing, and the difficulty of matching the throughput of a blockchain single chain structure due to the complexity of the grain food supply chain links, the large number of participants, and the large amount of data information, this paper proposes a grain food blockchain traceability information management model based on the master-slave multichain structure by analyzing the processes and data characteristics of each link in the grain food supply chain; on this basis, the PLEW consensus algorithm based on Raft + improved PoW algorithm is designed for the master chain, and the CI-PBFT consensus algorithm based on trusted information degree is designed for the slave chain. The master chain and slave chain are anchored to each other through hash locking, and the data is uploaded and queried through smart contracts. In order to verify the effectiveness of the model, the blockchain traceability system is designed and implemented based on Hyperledger Fabric2.2. At the same time, it is compared with the transaction throughput and traceability efficiency of the blockchain single chain structure. Through the safety analysis of the data information of a company in Hubei, the results show that the grain traceability system designed and implemented in this study has certain advantages over the blockchain single chain structure in all aspects. It can also solve the grain food security problems that consumers worry about, and provide reference for the research of grain blockchain traceability information management.

## 1. Introduction

With the growth of the world economy, the grain food culture, consumption culture and shopping demand of people's life are also gradually rising. At the same time, under the environmental background of the new coronal pneumonia epidemic, the safety and health incidents caused by the frequent occurrence of global grain food security problems, and the problems caused by the inability to track down defective products have also attracted people's attention. Grain food includes summer grain, early rice, and autumn grain according to harvest season, and products such as grains, potatoes, and beans according to crop varieties. According to China Economic Weekly, the global grain production in 2021 was 2.8 billion tons. China accounted for the largest proportion of 1365.7 billion kg, an increase of 26.7 billion kg over the previous year, or a 2.0% increase. The annual grain output reached a new record high and remained above 1.3 trillion kg for seven consecutive years [1]. For the people of China and even of the world, grain food can not only meet the basic needs of the human body but also prevent and assist the treatment of many diseases, including bowel cancer, appendicitis, and diabetes [2], so it is favored by the masses. However, the grain food is prone to mildew and deterioration in its storage [3]. In recent years, heavy metals in cadmium rice and fragrant rice exceed the standard [4], which greatly affects people's health and quality of life. Under the background of frequent grain food security problems, an efficient and comprehensive information traceability method is urgently needed to solve the problem.

The term “traceability” was first established and improved by the European Union in 1997 in response to the “mad cow disease” problem. In recent years, due to the frequent occurrence of grain food safety problems in the world, the research on the term “tracing” has become mature both at home and abroad [5, 6]. The traditional traceability technology is mainly through two-dimensional code or barcode technology, combined with radio frequency identification (RFID) technology, and through the use of information collection technologies such as the IOT in the physical layer to collect data. Through manual recording and storage in a centralized database [7, 8], consumers can trace the basic information of the product by scanning the barcode on the product package [9]. However, the process of grain food from farmland to dining table mainly includes five links: production, processing, storage, transportation, and sales. In addition, the data information of the company responsible for each link is complex, the format of data information among departments is not unified, and the data sharing is poor. The adoption of traditional traceability technology and centralized database will lead to low pellucidity of data information, reduced government supervision, and data is vulnerable to tampering by criminals [10, 11]; existing problems of traditional traceability are now one of the reasons why grain food security problems occur frequently [12].

In the early days, blockchain technology was considered as a kind of chained data structure composed of data blocks in chronological order and guaranteed by cryptography to be tamper proof and unforgeable distributed ledgers [13]. At present, blockchain technology is called a new computing paradigm by many scholars. It stores and verifies data information by using chain data structures, generates and updates data by using a distributed point-to-point consensus mechanism, uses cryptography to protect data security, and programs through intelligent contracts [14]. With the decentralization, distributed storage, data transparency, and traceability of blockchain, many scholars have applied it to the grain food industry in recent years. Ding designed a grain food security traceability system which adopted blockchain technology to solve the security problem of data storage [15]. Tao et al. put forward a comprehensive evaluation model based on blockchain smart contract technology, which improved the credibility and security of the grain industry through automatic detection of smart contracts [16]. Mao et al. proposed a credit evaluation system based on blockchain smart contracts, which improved the effectiveness of food supply chain supervision [17]. To sum up, the application of blockchain technology in the grain food traceability scheme can effectively solve the problems of low transparency of grain food traceability data information, and can also reduce the government supervision and tamper data easily. However, at present, most of the blockchain technologies applied to the grain food traceability scheme are blockchain single chain structure. With the increase of data in all links of the grain food supply chain, problems emerged in the blockchain single chain structure applied to the grain food traceability model, such as low consensus efficiency and low transaction throughput.

Given the existing problems in the blockchain single chain structure above, some scholars have designed blockchain multichain structure for various industries in the past two to three years. Zuo et al. proposed a privacy protection K-means clustering algorithm under blockchain multichains. This algorithm uses the idea of homomorphic encryption to achieve K-means clustering under multichains, to solve potential collusion attacks and eavesdropping attacks, and to avoid data leakage [18]. Zhou et al. designed a multichain blockchain dynamic partition method for microgrid power trading, which can not only maximize the total amount of power trading per unit time, but also achieve decentralization [19]. Liu et al. proposed a two-level branch structure blockchain expansion model, and the experiment proved that the two-level branch structure expansion model has great advantages in data storage efficiency and network load [20]. Zhang et al. designed a traceability model for the whole grain, oil, and food supply chain by using blockchain and identification technology. The model uses the data multimode storage mechanism to provide an optimization scheme for grain and oil food traceability [21]. To sum up, the research on the multichain direction of blockchain can solve the problems of low consensus efficiency, low transaction throughput and high delay in the blockchain single chain structure. Therefore, relying on blockchain based multichain structure, this paper proposes a grain food blockchain traceability information management model based on the master-slave multichain structure to solve the problems of the current blockchain single chain structure.

For the research of this paper, we have conducted relevant work in the early stage and referred to a large number of literature [21] for details. The structure of this paper is as follows: firstly, in the relevant work part, the application of blockchain single chain structure in grain food traceability industry in recent years is studied, and then the application of blockchain multichain structure is analyzed and compared. Secondly, in the part of grain food blockchain traceability information management model of master-slave multichain, the supply chain links and key information of grain food are specifically analyzed. According to the complex characteristics of different supply chain links and information redundancy, the overall framework of the model is designed, and a data storage model of blockchain master-slave multichain structure is designed based on the overall framework. In addition, a grain food traceability information management model based on the blockchain is built with the blockchain technology. In order to improve the overall traceability efficiency, a PLEW consensus mechanism is designed for the blockchain main chain, a CI-PBFT consensus mechanism is designed for the blockchain slave chain, and relevant data transmission and query smart contracts are designed. Thirdly, in the analysis part of the results, the theoretical verification analysis of the model is carried out first, and the feasibility analysis and comparative analysis are used to verify the feasibility of this model. Then, the system is built through the Hyperledger Fabric2.2 framework, and compared with the transaction throughput and consensus efficiency of the blockchain single chain structure. After that, the security performance of this model is analyzed through an example. Finally, the conclusion summarizes the full text.

## 2. Related Work

Over the past years, with the arrival of blockchain version 2.0 and web 3.0, more and more scholars tend to combine blockchain technology with grain food traceability supply chain to design a safe and convenient traceability system. However, there is little research on applying the blockchain multichain structure to the grain food traceability industry.

Blockchain obviously means a chain composed of one block after another. Each block stores certain information, which is connected into a chain according to their respective time sequence. It has the characteristics of decentralization, independence, being not easy to be tampered with and traceability. Blockchain multichain adopts the new scheme of “one chain, one contract” to redesign a public chain to ensure the normal operation of each contract. The blockchain multichain structure can ensure that the traffic surge on one chain will not affect the efficiency of the other chain, and any business conducted on the chain will not be interfered by other businesses, effectively realizing resource isolation. For the blockchain master-slave multichain structure, it means that the master chain is the first chain generated by the blockchain system, and the slave chain is the extension chain of the master chain. It has the characteristics of the blockchain multichain structure. The data growth between the master chain and slave chain will not affect each other, and the master chain and slave chain serve each other.

Nowadays, a lot of scholars use blockchain technology to solve related food application problems. Some cutting-edge research based on blockchain technology applied to the food industry is shown in Table 1.

First, based on China's influence on blockchain policies, some scholars have analyzed and studied policies under different supply chains. Liu et al., based on the national subsidy and incentive policy support for the blockchain [22], set different supply chains as research objects in the traceability service [23], agriculture, and [24] low-carbon fields in the blockchain field, set demand functions according to the characteristics of their respective fields, and analyzed the corresponding subsidy models. Their research provides theoretical guidance for the government to formulate and implement subsidy strategies. This enables the state to provide more support for blockchain technology.

At present, lots of students have applied blockchain technology to the grain food traceability industry and designed different innovative ways. Lin et al. proposed a blockchain based security mutual authentication system BSeln, which implemented fine-grained access control policies [25]. The system provides privacy and security guarantee for data information with auditability and confidentiality, reflecting the security and reliability of the blockchain. He and Hu designed a food cold chain traceability system based on blockchain technology, and used quantum secret key distribution technology to replace the asymmetric encryption technology of traditional blockchain, effectively improving the security of data information storage in the traceability system [26]. Dong et al. designed a grain and oil food reliable traceability model which used the blockchain technology, and replaced the centralized database with “on chain + cloud database.” This storage model can solve the low efficiency problems caused by the blockchain single chain structure [30]. Yang et al. designed a vegetable traceability system based on “blockchain + RFID technology,” which can solve some problems in information collection by using RFID (radio frequency identification technology) [31]. However, due to the increasingly complex data information level and the increasing number of participants, the single blockchain has been unable to solve the problems of low consensus efficiency and transaction efficiency reduction. For blockchain multichain structure, most mathematicians currently apply it to other industries. Xuan et al. proposed a distributed energy trading method based on multichain collaborative blockchain, which solved the problem of slow transaction speed by adopting blockchain system according to the method of parallel transaction processing from chain according to regional partition [37]. Li et al. proposed a new algorithm. The blockchain system is based on CPS storage under multichain edge cloud computing, in which the nodes of the system are divided according to the close communication relationship, and the time and space of data synchronization can be reduced through the partition storage structure of this algorithm [32]. Zhang et al. designed an interoperable multiblockchain reliable tourism management platform based on integrated REST API. The platform references the structure that uses multiblockchain to improve processing capabilities and help the tourism industry to add various commercial services [38]. It can be seen that the blockchain multichain structure has gradually become the research trend of blockchain application in all walks of life.

However, there are few studies on the application of blockchain multichain structure to the grain industry, but some scholars also use the multichain structure to study the traceability and control of grain. Peng et al. designed a multichain cooperation model based on blockchain + subchain to help rice supply chain achieve safe and reliable management and control [34]. Yu et al. designed a grain food traceability model based on the blockchain multichain structure, which can achieve real-time control of the traceability data ledger and interchain transaction records, providing reference for the research of the agricultural blockchain traceability supervision system [39].

To sum up, the application of blockchain technology in the food traceability industry has become mature at present. However, since the traditional blockchain single chain structure has the characteristics of distributed storage and is not easy to be tampered with, it cannot solve the problem that the food industry's data information is jumbled and the consensus efficiency and traceability efficiency are reduced due to its complex hierarchy, as well as the numerous participants and long life cycle. For the blockchain multichain structure, its multichain consensus interchain consensus needs to be further studied. Therefore, this research makes use of the characteristics of blockchain distributed storage, decentralization, being not easy to be tampered with and anticounterfeiting, and combines the hash locking method to design a blockchain master-slave multichain storage structure, and divides each link of the grain food supply chain into five major links: production, processing, storage, transportation, and sales for data classification, and classifies it into shared information and encrypted information to ensure the security of data information. On this basis, two improved consensus mechanisms are designed for the blockchain master-slave multichain structure and applied to the blockchain master-slave chain to improve its consensus efficiency. Compared with the existing multichain structure, the master-slave chain structure can perform traceability queries more quickly and accurately by setting priorities. Finally, comparing the single chain structure with the master-slave multichain structure can effectively reflect the advantages of the master-slave multichain structure. It can provide reference for building a safe, reliable, and efficient grain food traceability system. Through comparing with existing research, this paper makes the following contributions:

1. It analyzes the links and key information of grain food supply chain and divides them into shared information and encrypted information to provide reference for other scholars
2. This paper designs a blockchain master-slave multichain structure, which provides solutions to the problems of inefficient and low throughput of blockchain single chain structure caused by miscellaneous data
3. This paper designs two different consensus algorithms, PLEW and CI-PBFT, respectively, for the master chain and slave chain, providing reference for improving the consensus efficiency of the master-slave multichain structure
4. The grain food blockchain traceability information management model based on master-slave multichain proposed in this paper is also applicable to other industries

Based on the research of literature [21], this paper first classifies the data information of the five links of the data grain supply chain into shared information and encrypted information, so that the data information queried by enterprises on the supply chain can be classified, and the specific blockchain network created will be displayed specifically, and the consensus algorithm between the master and slave chains will be improved to enhance the traceability efficiency of the overall model. While designing relevant data judgment, upload query of the smart contract. Finally, the system functions were improved, and the supervisor login module was added to query blockchain transaction information.

## 3. Grain Food Blockchain Traceability Information Management Model Based on Master-Slave Multichain

### 3.1. Grain Food Supply Chain Links and Key Information Analysis

This section takes the grain food supply chain with frequent grain food safety problems as an example. Readers can refer to this example to analyze other industries. In the food supply chain, there are many participants, which can be divided into internal participants and external participants according to their participation methods. The internal participants are mainly farmers, processing enterprises, storage enterprises, logistics transportation enterprises, and sales enterprises. External participants mainly include consumers, government, and other regulatory departments, as well as quality inspection departments [27]. Moreover, the data in the whole grain food supply chain is complex, including shareable information and encrypted information. The information that can be shared is the information that can be shared for consumers and related enterprises in the supply chain, including basic information of grain food products, basic information of all links, environmental information, and staff information. Encrypted information is private data information shared within the enterprise, including product quantity, product cost, sales price, and data information generated during specific transactions [33].

In this study, the grain food supply chain is divided into five main parts, namely production, processing, storage, transportation, and sales. The planting link refers to the operation of seed selection, seed soaking, seedling raising, transplanting, fertilization, harvesting, and other operations for grain seeds and the recording of key information, such as seed and achievement information, environmental information, and transaction information. Processing link refers to the process of peeling, milling, extracting, refining, and other processes of harvested grains and recording key information, such as product information, processing link, quality inspection results, and processing costs. The warehousing process refers to the storage of processed products to prevent the damage, mainly recording hinge information, for example, warehousing links, locations, warehousing time, quality inspection methods, warehousing costs, and others. The transportation process refers to the transportation of the well preserved products in the warehouse to the destination, and the recording of their logistics information, logistics cost, and other crux information. The sales link refers to selling products and recording key information such as sales information and transaction information. We recorded relevant sales information, transaction information, and other key information, as shown in Table 2.

### 3.2. Overall Framework of the Model

Under the existing blockchain single chain structure, each node needs to store all the information on the chain. There are problems in its capacity, performance, running speed, and information security, which cannot meet the needs of customers on the alliance chain at this stage [40]. To resolve the questions of blockchain single chain, Ethereum 2.0 regional scheme and Hyperledger Fabric 1.0 multichannel scheme are extended to multichain structure on the basis of blockchain single chain structure, and the nodes on each chain only need to store the data information of the channel, solving the problem of low load of single chain [41, 42].

**Figure 1:**
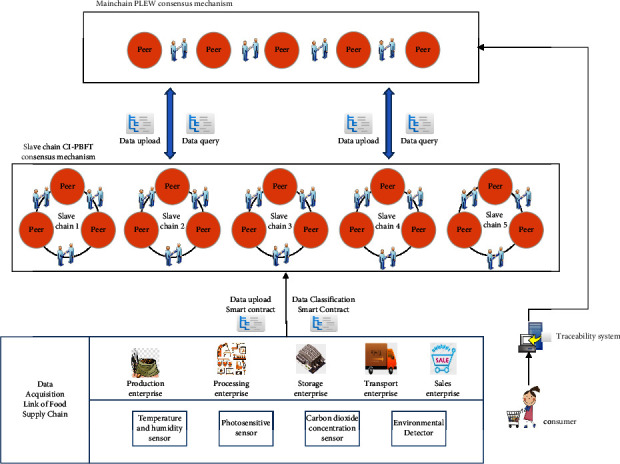
Grain food supply chain information management model based on blockchain master-slave multichain.

Figure 1 shows the overall framework of the grain blockchain traceability information management model based on master-slave multichain designed in this research. After collecting data information from all taches of the grain food supply chain through automation devices such as the Internet of Things (IoT), the model uploads data information to the blockchain network with Hyperledger Fabric as the underlying structure through data classification and uploading smart contracts. To guarantee the safety and reliability of encrypted information and shared information in the grain traceability model and solve the problem of miscellaneous and diverse information in each link, this research adopts a master-slave multichain structure model based on the blockchain single chain technology. The definition of master-slave multichain is as follows: main chain: the first chain generated by the system, responsible for the confirmation of the slave chain to ensure that the slave chain can operate well. Slave chain: a blockchain created by extending the main chain with side chain technology. If the chain is the extension of the master chain, it is called the primary slave chain; if it is the extension of the primary slave chain, it is called the secondary slave chain, and so on. The extended chain is called the parent chain, and the extended side chain is called the child chain. A parent chain can have multiple child chains, while a child chain can only have one parent chain. This storage model constructs a blockchain slave chain for each link of the supply chain to store the data that needs to be stored in this link. A total of five slave chains are designed according to the five major links of production, processing, warehousing, transportation, and sales. The blockchain master chain stores the index information and hash value and other data information of the slave chain. Because the master chain stores less data, only one master chain needs to be created. Cross chain processing between the master chain and slave chain uses hash time locking technology to anchor each other. Hash time lock includes two parts: hash lock and time lock. Time locking in master-slave multichain refers to the transaction between master chain and slave chain, which is valid only when it is completed within the specified time, and it is invalid if it exceeds the time (whether it is master chain or slave chain). Hash locking means that for a hash value *H*, if the provided *R* makes Hash (*R*) = *H*, the commitment is valid, otherwise it becomes invalid. Under the blockchain master-slave multichain structure, the master-slave chain is anchored to each other by means of hash time locking. If the data information cannot be queried or uploaded successfully for various reasons, the time lock and hash lock can prevent the data information from being stolen by criminals. Based on the characteristics of decentralization, decentralized network, traceability, consensus mechanism, security, high availability, tamper proof, and programmability of blockchain technology, and by combining with the links of grain food supply chain such as planting, processing, warehousing, transportation, sales, and supervision, the corresponding nodes in the blockchain network is built. By using the master-slave multichain storage model based on blockchain to manage the traceability data, a grain food supply chain traceability information management model is built to realize the supervision of the whole grain food supply chain.

Nowadays, the peer in the alliance chain are provided by each institution with several nodes, and each node is interconnected to form a distributed network in which transactions and blocks are spread and agreed. In addition, each node in the federation chain can apply to join a channel and communicate with other nodes in the channel after authentication and certificate sending. This paper comprehensively compares the consensus algorithms in the alliance chain, and finally adopts the practical Byzantine fault-tolerant (CI-PBFT) consensus algorithm for data consensus in the secondary chain by adding credit information. Since the main chain is a traceability inquiry chain, it is not necessary to store a large amount of data. The amount of data stored in each link of the grain food supply chain is different, so the main chain uses the PLEW (improved PoW + Raft algorithm) consensus algorithm. The specific two algorithms and how to operate between the master and slave chains are described in the following sections.

### 3.3. Master-Slave Multichain Storage Model Principle

**Figure 2:**
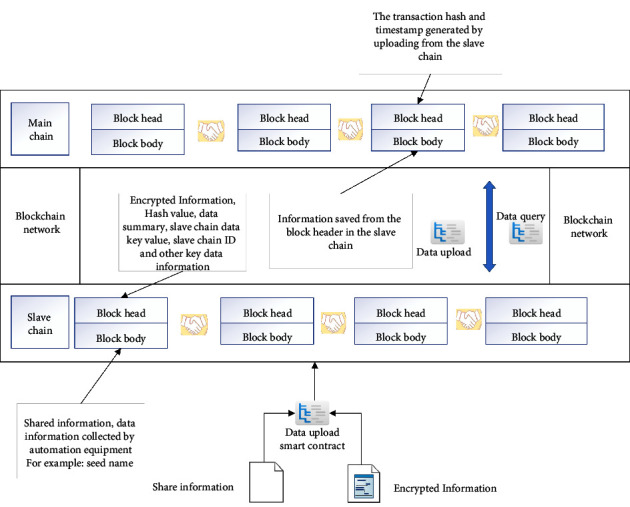
Master-slave multichain storage model.

This section introduces the principle of the blockchain master-slave multichain storage model in the production link. Other taches in the supply chain can refer to this principle. As shown in Figure 2, when the data information collected by the automation equipment in the production process needs to be uploaded to the network, first it is needed to classify the data into shared information and encrypted information through data classification, and then upload the data information to the blockchain slave chain through the data upload smart contract. Key data information such as encryption information, hash value, data digest, and slave chain ID are stored from the block header in the slave chain, and shared information and other data information collected through automation equipment are stored in the block body, such as seed name and planting time. The data verified and uploaded by the smart contract will be broadcast in the network from the slave chain. The consensus node will send the data information to each node in the consensus, and each node will store the verified data in the distributed ledger. Meanwhile, the consensus node will transfer the key data information from the slave chain block to the main chain through the data upload smart contract. The block header in the main chain of the blockchain stores the transaction hash, timestamp, and other information generated during the slave chain transaction, and the block body stores the data information in the slave chain block header.

The transactions between the master chain and slave chain connect the slave chain and the master chain through the hash locking method. In order to ensure that the data information on the blockchain is safe and reliable, the production to sales enterprises on the food supply chain will protect the security and reliability of the main chain of the blockchain, while the government and other regulatory authorities will protect the security of the slave chain to prevent illegal elements from tampering with and destroying the data information. In the master-slave multichain structure, the master chain, as a query chain, is responsible for querying and supervising the corresponding data information, and the slave chain, as a storage chain, is responsible for storing the data information of the food supply chain from production to sales.

The shared information can be queried in real time. The encrypted information can be queried only after the corresponding certificate is issued from the supply chain enterprise on the chain. In the master-slave multichain, the slave chain will package and upload each block to the master chain. After the slave chain block is authenticated by the consensus mechanism of the master chain, it will become an unbranched master chain block. Any criminal who wants to change the main chain block will have to pay huge costs on humans, materials, and financial resources.

### 3.4. Master-Slave Chain Consensus Algorithm

In this section, in order to make the master-slave multichain storage model more suitable for the grain food supply chain traceability information management model, two different consensus algorithms are designed for the blockchain master chain and slave chain, respectively, to ensure the timeliness of the master-slave multichain storage model. The two algorithms will be shown in the model analysis in Section 4.1.

#### 3.4.1. Slave Chain Consensus Algorithm CI-PBFT

**Figure 3:**
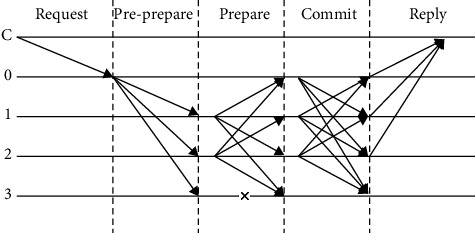
Consensus flow chart of byzantine fault-tolerant algorithm.

The existing blockchain consensus algorithms integrate alliance chain, public chain, and private chain such as PoW, PoS, DPoS, Raft, PBFT, and other consensus algorithms. The PBFT consensus algorithm is independent from digital currency and has low communication complexity. In addition, PBFT can accommodate the existence of faulty nodes and malicious nodes under certain conditions, providing security or flexibility for the system.  Figure 3 is a complete PBFT algorithm flow chart. There are four nodes numbered 0, 1, 2, and 3 in the system, with 0 as the primary node, 1, 2, and 3 as the replica node, and C as the client:

1. Client C sends a request to master node 0
2. The master node 0 assigns an integer number to the request, and then broadcasts the message content containing the integer number to other replica nodes
3. After receiving the message, the replica node sends a broadcast message to other nodes except itself
4. All nodes broadcast throughout the network to confirm the assignment serial number of the master node
5. Reply *C* to each node's confirmation message
6. Client *C* judges that a reply has been received and confirms the result

As this model is a grain traceability model, it has high requirements for the safety and credibility of data information, so on the basis of the original PBFT consensus algorithm, CI (trusted information degree) standard is added, and the slave chain uses CI-PBFT consensus algorithm to resolve the questions of information safety. If the information uploaded to the slave chain is malicious, the speed and success rate of information transmission can be greatly reduced by setting the trusted information, so as to prevent criminals from malicious upload. Suppose that there are *m* byzantine nodes among participating nodes CI-PBFT consensus algorithm, and the sum of all nodes is *N*. For all nodes, they are divided into consensus node CN (Consensus Node) and preset node PN (Preset Node) in a certain proportion. The consensus node participates in the consensus process, and the preset node saves the consensus results as a successor node but does not participate in the consensus process. Before the CI-PBFT consensus starts, all nodes are ordinary nodes. If a node wants to enter the consensus node group, it needs to submit an application and broadcast identity registration to the whole network. When the node passes the review, it will enter the consensus node group. If there are too many CN nodes, the whole consensus process will be affected, while too few will affect the consensus results. Therefore, in comparison, 70% of the nodes are selected to form a CN cluster, and the remaining 30% are taken as PN clusters. When the CN nodes meet their set threshold, the other common nodes enter the PN cluster according to the sequence number of entering the network. Set CN node cluster as *C*_CN_ and PN node cluster as *C*_PN_, both of which should meet as follows:

(1) $$\begin{array}{c} N=C_{CN}+C_{PN}\text{.} \end{array}$$

In the consensus process, all common nodes in the initial state have 0 points. In a round of consensus process, when the consensus master node initiates a consensus and the consensus slave node is verified, the master node has +1 points and all slave nodes have +1 points. When the consensus master node is down or the consensus fails due to a malicious node, the master node that initiates a consensus request has 2 points less. When the integral is reduced to a negative number, it will automatically exit the consensus and be supplemented by the preset node. The CI-PBFT consensus algorithm can effectively reduce the occurrence of malicious data uploaded by criminals. CI-PBFT consensus algorithm will be shown in Section 4.1.

#### 3.4.2. Main Chain Consensus Algorithm (PLEW)

As a query chain, the main chain is proposed to use the PLEW (Raft + improved PoW) consensus algorithm. Nodes independently select several hash algorithms to perform PLEW calculation based on their own computing power and other resources, and add the successful results to the block. Because the amount of data uploaded in each supply chain stage is different, the workload is also different. The contribution degree of each phase is proved according to its workload (query amount), and the query priority is given according to its contribution degree. The workload proof algorithm (PoW) makes the Hash value of the content after the transaction data is hit meet the specified upper limit by calculating a value (nonce). After the node successfully finds the satisfying Hash value, it will immediately broadcast the package block to the whole network. When the network node receives the broadcast package block, it will immediately verify it. If the verification is passed, it indicates that some nodes have successfully solved the puzzle, and they will no longer compete for the current block packaging, but choose to accept this block, record it in their own ledger, and then perform the next block's competition puzzle. Only the fastest puzzle solving block in the network will be added to the ledger, and other nodes will copy, thus ensuring the uniqueness of the entire ledger. If any node cheats, it will cause the node of the network to fail the verification, and directly discard its packaged block, which cannot be recorded in the general ledger. The cost of the cheating node is huge and useless, so users on the chain will consciously abide by the consensus algorithm, ensuring the security of the main chain.

However, since the time consumed by the PoW consensus algorithm is related to its mining difficulty, its mining difficulty is not in the block information, but only depends on the rule dynamic calculation in the network node. Its rule formula is shown in formula (2), where *T* is the abbreviation of Target and *D* is the abbreviation of Diversity. *T*_1_ and *T* are large numbers with 256 bits, where *T*_1_ is a very large constant 2^256−32^ − 1 = 2^2^24 − 1. According to the formula, *T* is the abbreviation of target and the greater the difficulty of *D*.

(2) $$\begin{array}{c} D=\frac{T_{1}}{T}\text{.} \end{array}$$

However, this is a traceability query system. If it takes a long time to simply use the PoW formula algorithm, it will have a lower traceability timeliness. Therefore, PLEW consensus algorithm, which combines Raft consensus algorithm with the improved PoW consensus algorithm, is used to improve the low timeliness of PoW.

**Figure 4:**
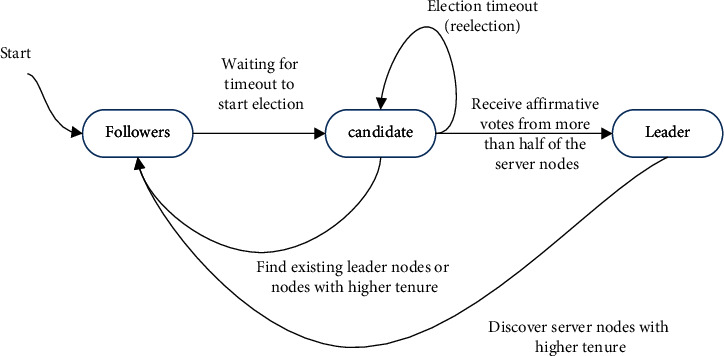
Server state transition model.

Raft consensus algorithm requires two roles, the leader and the follower. There are three states in the node, namely, the leader, the candidate, and the follower. The leader is responsible for transmitting client instructions, and the follower is responsible for executing the leader's commands. The transition of these two roles requires an intermediate role, so Raft needs three states: the follower, the candidate, and the leader. The three state process transitions are shown in Figure 4.

The PLEW consensus algorithm proposed in this paper firstly selects the leader node through Raft consensus algorithm. The leader node is the node whose query times are more than 30% of the total query times. Secondly, the leader node is optimized through the improved PoW algorithm to improve the timeliness of the main chain. The improved PoW algorithm reduces the computing power required to find random numbers by reducing the difficulty of hash calculation. The initial difficulty is set to 1, and the corresponding difficulty value is 3, that is, the first three bits of the hash value obtained by splicing the hash value of the previous block with the found Nonce are 0, so it takes less than 4 s to find the results. For the traceability model, it not only ensures the security of data information, but also ensures the timeliness of traceability.

### 3.5. Design of Smart Contract

**Figure 5:**
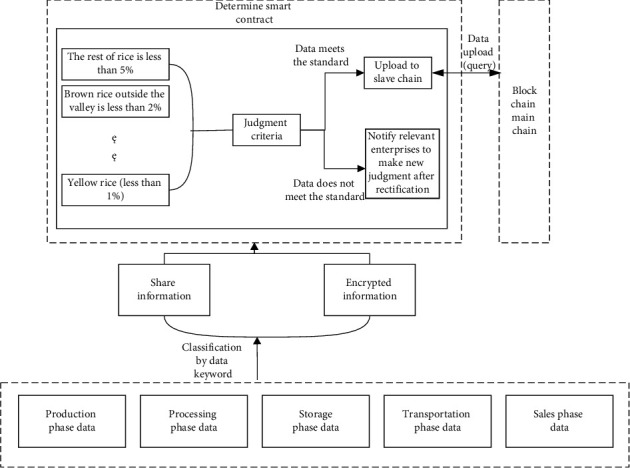
Flow chart of smart contract.

To sum up, it is a computer protocol that achieves information dissemination, verification, and contract execution through programming code [35], and contract participants can participate in modifying these protocols. Smart contract is an important symbol of blockchain 2.0, which makes blockchain expand from digital currency to other fields [28, 43]. Smart contracts have the characteristics of security, order, verifiability, decentralization, and automatic execution on the blockchain network [44], which can be used to the traceability system of the grain food supply chain to effectively improve its shortcomings of lengthy and slow traceability. Food supply chain data information is complex and there are many links. In its lengthy data traceability stage, food safety and health accidents are easy to occur, and it is very difficult to find the cause of the accident. Therefore, smart contract technology can be used to monitor the data of each link of the food supply chain, so that it can quickly find the source of the accident, so as to improve the frequent occurrence of food health security. As shown in Figure 5, this smart contract performs algorithm analysis to judge the uploaded data according to the national standard of the People's Republic of China-Rice GB1350 (Safe Storage and Quality)-2009. If the uploaded data does not meet the standards, the relevant enterprises will be notified to rectify through the smart contract in a timely manner, and the judgment will be made again after the rectification. If the data meets the standards, it will be directly uploaded to the slave chain. The smart contract for data determination is shown in Algorithm 1. When data is uploaded to the slave chain, the master chain, and slave chain will anchor each other through hash time locking, and upload and query data through the data upload and query smart contract. The smart contract algorithm for uploading data information from the chain to the main chain is shown in Algorithm 2.

In Algorithm 2, Hash (*R*) = *H* and time are prerequisites for hash time locking.

## 4. Results and Discussion

### 4.1. Model Analysis

#### 4.1.1. Feasibility Analysis

**Figure 6:**
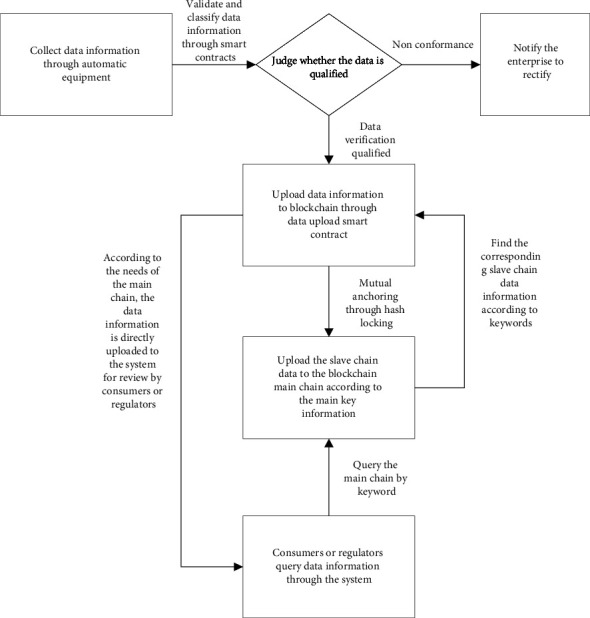
Flow chart of model operation.

The operation flow chart of grain food blockchain traceability information management model based on master-slave multichain is shown in Figure 6. By virtue of the automatic equipment and manual recording, when the information in the grain food production link such as the seed source, name, planting environment, and planting cost, the information in the processing phase such as the product, processing environment, quality inspection method, and quality inspection result, the information in the warehousing link such as the storage location, warehousing method, and warehousing cost, the information in the transportation link such as the transportation mode, departure place, destination, and storage cost, as well as the information in the sales link such as the sales time, place, and price, are collected, and then in the data upload stage, the format and standard of the data are filtered through the smart contract verification, and they are classified according to their keyword information. Then, the data information is uploaded to the blockchain slave chain through the data upload smart contract, and their main key information is transferred to the blockchain mainchain through the smart filtering. When the consumer needs to query or supervise the data information of the product, the main chain calls the relevant data from the chain by querying the smart contract and directly transmits it to the system, effectively improving the efficiency of the traceability supervision data information.

According to the theoretical analysis of the model, the model will be systematically built in Section 4.2, and the model will be tested and analyzed according to the actual data validation of relevant enterprises to prove the feasibility of this model.

#### 4.1.2. Consensus Performance Analysis

In this part, the feasibility of the system is analyzed by designing the master-slave chain algorithm in the master-slave multichain model and applying it to the system built in Section 4.2. CI-PBFT consensus algorithm for slave chain is shown in Algorithm 3.

For the design of the slave chain CI-PBFT consensus algorithm, if the criminals want to tamper with and destroy the data information, their own trusted information will be reduced, and their nodes will also withdraw from the consensus. This algorithm can effectively guarantee the safety and credibility of record information, and reduce the number and success rate of the criminals' destruction of data information.  Algorithm 4 shows the improved PoW algorithm. The PLEW algorithm used for the main chain is shown in Algorithm 5.

Compared with the slave chain, the master chain, as a query chain, needs to grade the query users. For users who have many queries and visits, setting the priority of the leader node can make it easier for users to query, monitor data information and other operations, and provides theoretical support for model implementation.

Compared with the traditional PBFT consensus algorithm, the slave chain CI-PBFT algorithm proposed in this model not only extends the advantages of PBFT nodes in reaching consensus faster, lower latency, higher throughput, and energy conservation, but also adds the standard of credit information to reduce the frequency of illegal elements' destruction of data on the grain food supply chain and improve the security of data information. At the same time, compared with the traditional PoW algorithm, the PLEW consensus algorithm used by the main chain upgrades the timeliness of the traceability model by reducing the difficulty, and selects the leader node to determine the access priority according to the Raft consensus, effectively improving the timeliness of the traceability information management model.

### 4.2. System Design and Implementation

#### 4.2.1. System Overall Framework Design

**Figure 7:**
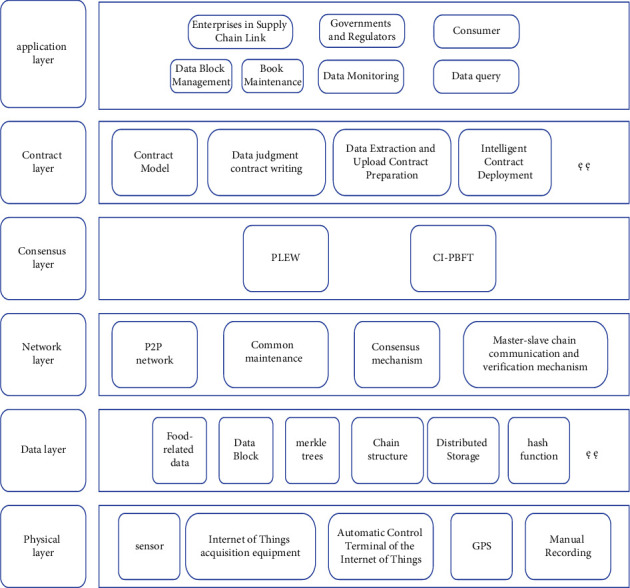
Overall framework of the system.

The grain food supply chain involves many subjects, which makes it more difficult to trace its products. The blockchain technology can provide the grain food supply chain with solutions that make it more difficult to trace its data and information. With the progress of science and technology and the increasing growth of people's living standards, the main body of the supply chain has become more complex, and the data information has become more diverse, and the traditional single chain structure has been unable to adapt to its operating speed, so the master-slave multichain structure is used to solve this problem. However, blockchain technology only solves problems such as preventing data from being tampered with and writing false information, but cannot conduct substantive data collection. Therefore, Internet of Things devices are cited as the information collection function of each link of the grain food supply chain to ensure the authenticity and reliability of data information [29]. Based on the blockchain technology and combined with the overall framework of the system involved in each link of the grain food supply chain, as shown in Figure 7, it is divided into six layers according to the function, namely the physical layer, the data layer, the network layer, the consensus layer, the contract layer, and the application layer.

The physical layer collects information on production, processing, storage, transportation, and sales of the grain food supply chain through IoT devices, sensors, GPS, and manual records. The data layer generates data blocks from the grain food related data collected by the physical layer, establishes distributed ledger storage data, encrypts the data through asymmetric encryption technology, and forms a chain storage structure through hash functions, so that the data cannot be easily tampered with, which ensures the security and reliability of the data [36]. The network layer encapsulates the networking mode (P2P network) of the blockchain system, data verification and propagation mechanism, and other elements. The consensus layer establishes the PLEW consensus mechanism adopted by the main chain and the CI-PBFT consensus mechanism adopted by the secondary chain. The contract layer mainly establishes a contract model through programming, writes contracts for judging data, extracting and uploading data, and automatically executes the contracts when the operation mechanism is met [45]. The application layer mainly provides information interaction services and ledger maintenance services for enterprises in the supply chain link through the visual terminal interface, provides data supervision services for the government and regulatory authorities, and provides front-end interface data query services for consumers.

#### 4.2.2. System Development Environment

The system uses the Hyperledger Fabric system under the alliance chain as the development platform. Since the alliance chain only targets members of a specific group and a limited number of third parties, it internally designates multiple preselected nodes as bookkeepers. The generation of each block is jointly determined by all preselected nodes. Other access nodes can participate in transactions, but do not interfere with the accounting process. Therefore, it can meet the requirements of multiple nodes and participants in the complex grain food supply chain. The traceability information management system includes the blockchain network of Hyperledger Fabric 2.2 underlying structure. The traceability system web front-end interface is developed using Python language and Django framework. The blockchain network is built on CentOS Linux 7.5 operating system based on the multiple docker containers built by the Fabric operating system. The required peers in the fabric network are deployed through the docker compose command, and smart contracts are written in the go programming language, The Docker version is 2.10.9, the docker compose version is 1.29.2, and the go language version is 1.19. The CoutchDB with the Fabric framework is used as the database to store data. Meanwhile, the network of blockchain uses the Fabric node sdk module to provide developers with pluggable APIs, user clients, network components required by the blockchain [46].

**Figure 8:**
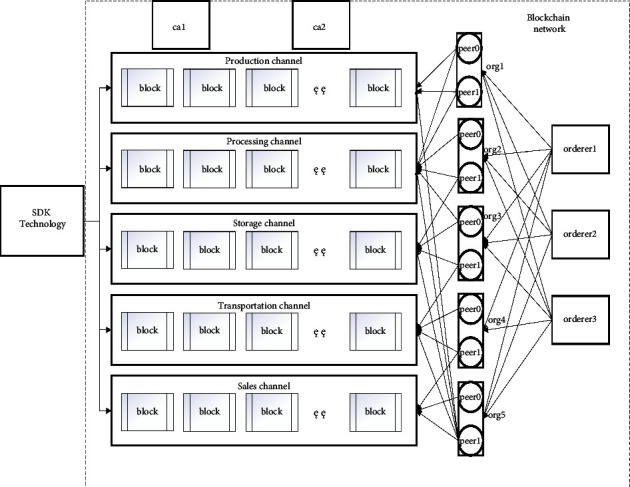
Deployment diagram of the blockchain network environment.

This system sets the five links of production, processing, storage, transportation, and sales as organizations and adds them to the blockchain main chain. The essence of organizations is permissions, and different permissions can be given to different links. Each enterprise builds a slave chain according to its needs, and sets up two related departments and one supervision department according to the enterprise needs, as shown in Figure 8. The blockchain network is deployed under the VMware virtual machine with multiple machines and peers. The virtual machine is configured with 64G memory, 16 core processor, and 200G hard disk. The five links of the whole grain food supply chain, namely, from production to sales, are set as five organizations, each organization contains two peers. The peer is essentially a data storage peer. To ensure the authority of the regulatory authority, three ordering nodes are added to the fabric network. The sorting nodes are the same as ordinary nodes. Different organizations can be granted different permissions by configuring certificates, ca1 and ca2 are certificates produced by five links and supervision departments.

#### 4.2.3. Systematic Implementation

**Figure 9:**
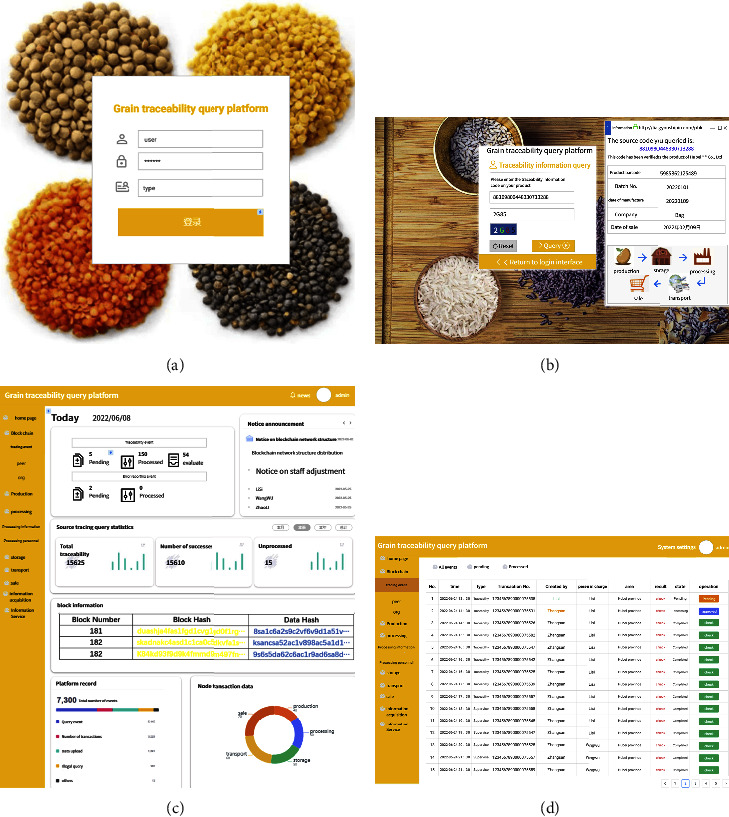
Traceability information query platform interface. (a) The login interface of the system. (b) The system query information interface. (c) and (d) The interface that can be queried after the supervision department or the supply chain department logs in.

This system takes the product supply chain of a rice limited company in Hubei Province of China as the tracing target. Rice is the largest grain crop in China, and Hubei Province is one of the provinces with the widest distribution of rice production in China. In the actual application of the system, the corresponding unique production batch number is generated according to the production and processing location of rice, environment, and other information. If it is checked that it meets the national food safety and health standards, it will enter the storage, transportation, and sales links. The traceability system interface is shown in Figure 9.

**Figure 10:**
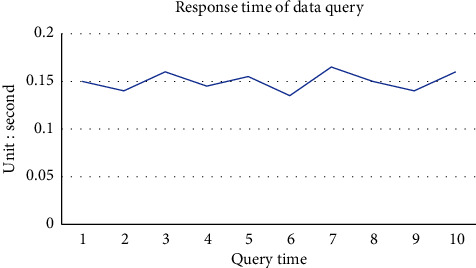
Traceability response time result chart.

The Web login interface of the traceability system is shown in Figure 9(a). Users log in to the system according to their own permissions. When the consumer user logs in to the system, the interface is shown in Figure 9(b). After entering the query code, the consumer can view the basic information such as the batch number and production date of the traceability product. At the same time, in order to verify the traceability query time, we query the traceability codes of different batches, and find that the average response time is 0.15 s, as shown in Figure 10. When the supervisor and the department on the supply chain log in, the interface is as shown in Figure 9(c). This interface can manage blockchain, production, processing, warehousing, transportation, sales, and other links, and view block information, personnel information, platform records, node transaction data, and other information.  Figure 9(d) shows the event data information generated recently. The event types include traceability and supervision. Supervisors and supply chain departments can view their specific data information.

### 4.3. System Test Results and Analysis

In order to ensure the normal operation of the system and its security performance, the system operation efficiency, throughput, and security performance are tested.

**Figure 11:**
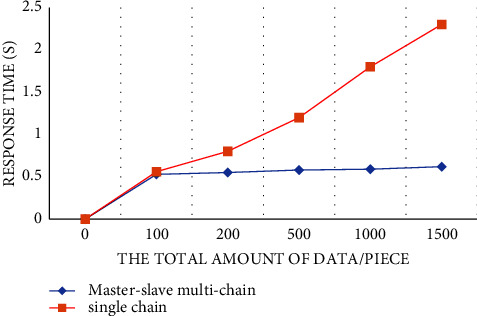
Comparison of query time between single chain and master-slave multichain.

**Figure 12:**
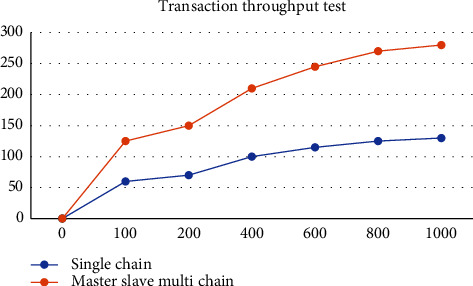
Comparison of single chain and master-slave multichain transaction throughput.

**Figure 13:**
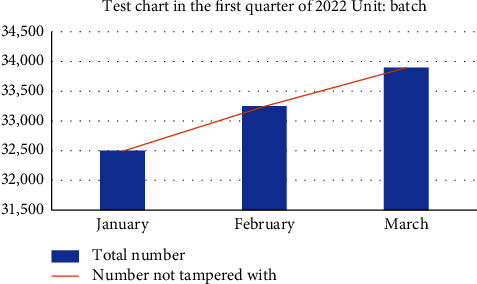
Data information security test chart.

In the system performance test, this research proposes a grain food supply chain traceability information management model based on the master-slave multichain structure, which protects the input and reading of encrypted information in all links of the supply chain through smart contract technology, and solves the data storage pressure caused by the lengthy and complex data information of the grain food supply chain under the traditional blockchain single chain structure [47]. The master-slave multichain structure model proposed in this study is that production, processing, storage, transportation, and sales each have a slave chain, and each slave chain has a desired node to store data, so the reading and writing of shared information and encrypted information are strictly controlled. In addition, in order to explore the performance comparison between the traditional blockchain single chain structure and the master-slave multichain structure, the PBFT consensus mechanism is adopted by designing and deploying a single chain blockchain network structure with five nodes, and the PLEW consensus mechanism is adopted by deploying a master chain with five nodes. Five slave chains with 10 nodes each adopt the master-slave multichain blockchain network structure composed of CI-PBFT consensus mechanism. When the total number of data is 100, 200, 500, 1000, and 1500, compare data information of 50 queries. It can be seen from the results that when the data query volume is less than 100, there is almost no difference in the query time between the two. But when the data volume increases to a certain extent, the efficiency of adopting a consensus blockchain single chain structure decreases significantly, and the query time increases gradually. The master-slave multichain structure uses different consensus mechanisms of the master-slave chain to ensure that the query time is basically unchanged. The test results are shown in Figure 11. As shown in Figure 12, in the transaction throughput test, the master-slave multichain structure has certain advantages over the single chain structure in terms of the transaction throughput between 50 and 1000. Meanwhile, to test the safety of the traceability system, the system performed safety verification with the data information of a wheat variety of a grain, oil and food company in Hubei Province. The test was conducted in the first quarter of 2022. During this period, the company set up wheat batches for 2132 times, and the traceability accuracy rate reached 100%, which shows that the accuracy of the system for traceability accuracy is extremely high. At the same time, the safety of data information storage of the blockchain master-slave multichain structure was tested. In the first month, about 32500 batches of data information were stored; in the second month, about 33250 batches of data information were stored; and in the third month, about 34350 batches of information were stored. The results are shown in Figure 13. The data shows that the number of malicious tampering with data information by criminals is almost zero, which provides a strong support for the safety of data information storage of the system.

The three comparative verification results prove that the master-slave multichain grain blockchain traceability information model proposed by this model has more advantages in data credibility, timeliness, and practicality, and has certain advantages over traditional traceability methods and blockchain single chain structure.

At present, lots of students have applied the blockchain technology to the grain food supply chain traceability and supervision industry. However, most of the current research is limited to the blockchain single chain structure, and the single chain structure has gradually failed to meet the traceability, low supervision efficiency, poor timeliness, security degradation, and other problems caused by the iteration of data information updates on the food supply chain. At the same time, some scholars have applied the blockchain multichain structure to the grain food traceability industry, but it is not yet mature. In order to reflect the advantages of the grain blockchain traceability information management model of master-slave multichain proposed in this paper, this paper compares it with other relevant documents, and the results are shown in Table 3.

Literature [30] proposed a trusted traceability model of the grain and oil food supply chain based on blockchain technology, and solved the problem of grain food traceability security by using the blockchain “peer + cloud database” storage mode. Literature [39] proposed a grain traceability model based on the blockchain multichain structure, and on this basis, established a multichain data storage structure, designed a network access mechanism based on regulatory authorization to build a chain, and realized prechain supervision of data and on chain control of traceability nodes through smart contracts.

According to security performance analysis, compared with literature [30], it uses the storage method of blockchain peer + cloud database to store data. When a large amount of data flows into the cloud database, if there are criminals to destroy it, a large amount of data will be tampered and flowed out, causing serious losses for enterprises. The blockchain master-slave multichain storage structure proposed in this study can ensure the security and reliability of data and prevent illegal elements from destroying. For the vulnerability of data information, compared with the multichain structure proposed in literature [39], this paper sets the master-slave multichain structure, and uses hash locking to anchor each other between the master and slave chains. If you want to attack the slave chain, you must attack the master chain first, and the cost of attacking the master chain is huge, which greatly reduces the possibility of being attacked.

In terms of model efficiency, compared with the single chain single consensus proposed in literature [30] and the multichain single consensus proposed in literature [39], the master-slave multichain model proposed in this study sets priority in terms of query and supervision, which improves the transaction throughput and timeliness of the traceability model. In addition, different consensus mechanisms are designed for the master chain and slave chain to adapt to the traceability model, effectively reducing the delay of this model.

In terms of scalability, this model is developed using the Hyperledger Fabric open source framework, while literature [30] uses cloud database and literature [39] uses interstellar system files, both of which are nonopen source structures. Therefore, this model has more development prospects in terms of resource consumption.

**Figure 14:**
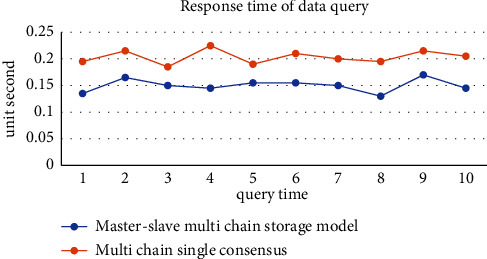
Traceability time comparison chart.

To prove the advantages of this model over the existing multichain research, we have analyzed the efficiency of the multichain single consensus and the multichain multiconsensus proposed by this model. In terms of data query time, the multichain single consensus costs more time than the multichain multiconsensus proposed by this model. The results are shown in Figure 14. It can be seen that this model has certain advantages over the existing multichain single consensus structure.

## 5. Conclusion and Prospect

This research proposes a grain food blockchain traceability information management model based on master-slave multichain. The blockchain master-slave multichain structure is used to store the data information of the five major links of grain food, including production, processing, storage, transportation, and sales. It effectively solves the problems of slow operation speed and reduced transaction throughput caused by the miscellaneous information under the traditional blockchain single chain structure. The master-slave chain is anchored through hash locking. It makes the data on the chain more difficult to be tampered with, improves the security and reliability of the data, and ensures the authenticity of the data on the traceability system. Moreover, on the basis of the blockchain master-slave multichain structure, in order to adapt to the traceability system, the PLEW consensus mechanism is designed for the main chain, and the CI-PBFT consensus mechanism is designed for the slave chain, which effectively improves the efficiency of traceability supervision data information and provides guarantee for the timeliness of the model. On this basis, Hyperledger Fabric2.2 alliance chain builds the system. The overall traceability information management model is of great significance for ensuring food quality and security and improving people's overall quality of life.

However, although most grain food enterprises use automatic equipment to collect and upload data information at present, a small number of small enterprises still upload data information in an artificial way, which makes it impossible to ensure the authenticity of product data information in the circulation process. This is also the deficiency of the design model of this research institute. The government and other regulatory authorities can conduct centralized agency certification and training for the input information personnel to reduce the harm of certification and dissemination of false information on the chain. In the future, we will combine blockchain technology with radio frequency identification technology (RFID) and other Internet of Things devices to guarantee the reliable collection of data information before the link, avoid too much human intervention, and design an algorithm protocol combining blockchain and RFID to guarantee the safety of data information during the upload process, so as to further better the reliability of the traceability system data information.

## Figures and Tables

**Algorithm 1:**
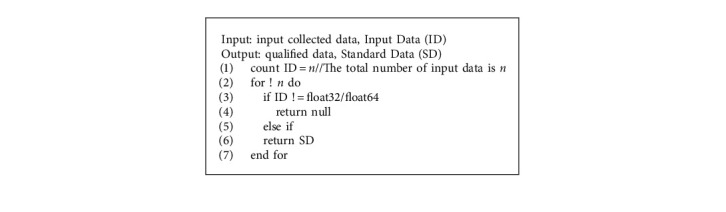
Data judgment smart contract.

**Algorithm 2:**
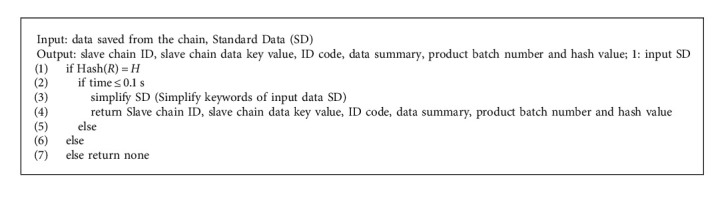
Data upload algorithm.

**Algorithm 3:**
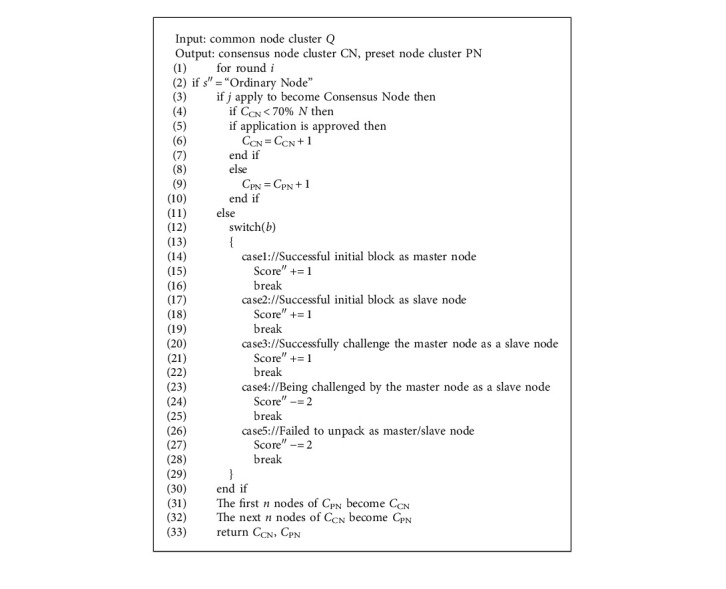
CI-PBFT consensus algorithm.

**Algorithm 4:**
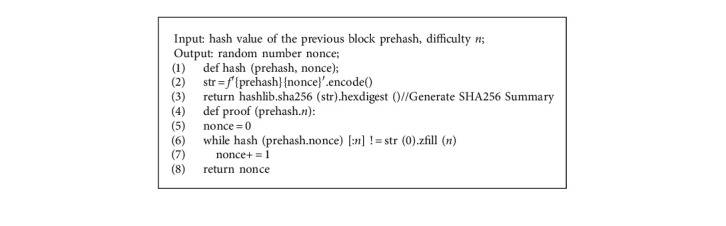
Improved PoW algorithm.

**Algorithm 5:**
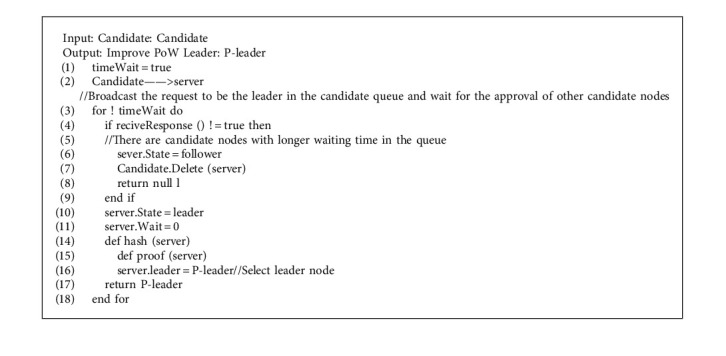
PLEW algorithm.

**Table 1:** Research overview.

Category	Research content	References
Blockchain policy related	Analysis of government subsidy policies through different supply chains	[22–24]
Blockchain based food or crop traceability solution	Solve the traceability problems of existing food or crops through blockchain technology	[5, 6, 9, 15, 21, 25–29]
Solutions to storage problems in food and other industries based on blockchain multichain structure	Solve the problems of low efficiency and low throughput caused by the increase of data information that cannot be matched by the single chain structure through blockchain multichain	[18–20, 26, 27, 30–33]
Solutions to food or crop traceability or supervision based on smart contract technology	Solve the problems of traceability or low regulatory efficiency and high cost through blockchain smart contract technology	[5, 16, 34–36]

**Table 2:** Key information of grain food supply chain.

Project	Production	Processing	Storage	Transportation	Sale
Share information	Seed source	Product information	Storage location	Type of shipping	Product information
	Seed name	Processing process	Storage method	Place of departure	Sales time
	Planting environment	Processing environment	Warehousing time	Transportation time	Sales method
	Planting time	Quality inspection method	Quality inspection method	Transport environment	Sales location
	Harvest time	Quality inspection results	Delivery time	Destination	Sales link

Encrypted information	Planting quantity	Product cost	Warehousing cost	Transportation quantity; transportation cost	Purchase price
	Planting cost	Processing expenses	Warehouse quantity	Transaction information	Selling price
	Transaction information	Transaction information	Warehousing expenses		Total products

**Table 3:** Comparison and analysis.

Performance	Index	Literature [21]	Literature [30]	This paper
Security	Data security	Middle	High	High
	Vulnerability	High	Middle	Low
	Timeliness	Low	Middle	High

Model efficiency	Throughout	Low	High	High
	Delay	High	Middle	Low

Scalability	Resource consumption	Middle	Lower	Low

## Data Availability

The data supporting this study are included within the paper.
